# Safety and efficacy of enterprise stenting for symptomatic atherosclerotic severe posterior circulation stenosis

**DOI:** 10.1186/s40001-023-01260-x

**Published:** 2023-08-17

**Authors:** Zhi-Long Zhou, Tian-Xiao Li, Liang-Fu Zhu, Li-Heng Wu, Min Guan, Zhen-Kai Ma, Yang-Hui Liu, Jin Qin, Bu-Lang Gao

**Affiliations:** Stroke Center, People’s Hospital of Zhengzhou University, Henan Provincial People’s Hospital, School of Clinical Medicine, Henan University, 7 Weiwu Road, Zhengzhou, 450003 Henan China

**Keywords:** Enterprise stent, Severe intracranial atherosclerotic stenosis, Posterior circulation, Symptomatic, Complications

## Abstract

**Purpose:**

To investigate the safety and efficacy of Enterprise stent angioplasty and risk factors for the prognoses in treating symptomatic severe posterior circulation atherosclerotic stenosis (SSPCAS).

**Materials and methods:**

Patients with SSPCAS who were treated with the Enterprise stent angioplasty were retrospectively enrolled. The clinical data, peri-procedural complications, postoperative residual stenosis, in-stent restenosis and recurrent stroke at follow-up were analyzed.

**Results:**

262 patients with 275 stenotic lesions treated with the Enterprise stent angioplasty were enrolled. The stenosis degree was reduced from 86.3 ± 6.2% before to 19.3 ± 5.4% after stenting. Complications occurred in 14 (5.3%) patients. Clinical follow-up was performed in 245 (93.51%) patients for 16.5 ± 7.3 months. During 1 year follow-up, 7 patients (2.9%) had recurrent symptoms, including 4 patients with stenting in the intracranial vertebral artery and 3 in the basilar artery. Imaging follow-up was conducted in 223 (85.11%) patients. In-stent restenosis was present in 35 patients (15.7%), with the restenosis rate of 26.4% (n = 23) in the intracranial vertebral artery, which was significantly (P < 0.001) greater than in the basilar artery (8.8%). Six patients (17.1%) with in-stent restenosis were symptomatic. The stenotic length was the only significant (P = 0.026 and 0.024, respectively) independent risk factor for 1 year stroke or death events and in-stent restenosis.

**Conclusion:**

The Enterprise stent can be safely and efficaciously applied in the treatment of symptomatic severe posterior circulation atherosclerotic stenosis, with a relatively low rate of in-stent restenosis and recurrent stroke within 1 year. The stenotic length was the only significant independent risk factor for 1 year stroke or death events and in-stent restenosis.

## Introduction

The stroke caused by posterior circulation atherosclerotic lesions accounts for 35% of all strokes in the posterior circulation [1]. Patients with severe vertebrobasilar artery stenosis are prone to recurrent cerebral infarction due to poor perfusion and unstable hemodynamics caused by the stenosis [2–5]. In addition, because the blood supply of the posterior circulation is concentrated in the area of conduction tracts and brain stem nuclei, small infarcts in this area may cause very serious neurological deficits [6], necessitating timely treatment of patients with posterior circulation atherosclerotic stenosis [7]. However, medical treatment alone is limited, and even with the best medication, the case fatality rate within 30 days after posterior circulation stroke has been reported to be 1.9%, with a severe disability rate of 18% and a mild disability rate of 51% [8]. Earlier studies did not support the use of intracranial stenting for atherosclerotic stenosis of large intracranial arteries because of the unfavorable 30 day rates of stroke or death and 1 year primary end points in the stenting group compared with the medication group [9, 10]. Nonetheless, recent studies have reported improved outcomes of intracranial stenting for intracranial atherosclerotic stenosis with a 2.6% periprocedural stroke, bleed, and death rate with the Wingspan stent [11] and an 8.5% 1 year stroke or death rate [12]. The latest CASSISS study has demonstrated comparable effects of stenting versus medication alone, with the incidence of stroke or death within 30 days being 5.1% in the stenting and 2.2% in the medication group, but no significant difference in the mortality during the 1 year follow-up period (8.0% in the stenting vs. 7.2% in the medication group) [13].

Currently, there are few published studies on symptomatic intracranial stenosis in the posterior circulation even though intracranial stenting has been suggested feasible for posterior circulation stenosis [14]. The safety and effectiveness of endovascular recanalization of stenosis at the vertebral artery opening have basically been made clear, but cases of intracranial vertebrobasilar stenosis will have to be carefully selected for endovascular intervention [15]. A relatively high perioperative complication rate of intracranial stenting (Wingspan stent) in the posterior circulation stenosis has been reported [10, 16, 17], and the ischemic events within 30 days were mainly perforator infarction, which may be caused by the Wingspan’s high radial force to injure the basilar artery [17]. In the treatment of intracranial atherosclerotic stenosis, the Wingspan stent has also been reported to result in a higher perioperative ischemic rate in the posterior circulation basilar artery versus in the anterior circulation (14.5% vs. 5.1%) as well as a higher recurrent stroke rate in the posterior circulation versus in the anterior circulation (10.4% vs. 3.4%) at 19 month follow-up [18]. These unfavorable events may be caused by the high radial force of the Wingspan stent as compared with that of balloon-expandable stents because sustained radial force leads to significant intimal hyperplasia [19].

Compared with the Wingspan stent (Boston Scientific, Boston, MA, USA), the Enterprise stent (Codman & Shurtleff, Raynham, MA, USA) has better flexibility and smaller radial force, which may help reduce perioperative complications [19]. The Enterprise stent has been reported in endovascular intervention of 189 patients with 209 intracranial atherosclerotic stenoses [20], with a technical success rate of 100%, a rate of major peri-procedural complications of 8.1%, a restenosis rate of 24.7% at 4.2 month follow-up, and a recurrent ischemic rate related to the stented artery of 2.2% at 10.2 months. In a study enrolling 35 patients with symptomatic severe basilar atherosclerosis stenosis treated with the Enterprise stent [21], the technical success rate was 100%, and the main procedural complication rate was 0, with a restenosis rate of 17.6% at 6 month follow-up and a TIA rate of 5.7% at 10.6 month follow-up. Currently, no large series of patients with posterior circulation atherosclerotic stenosis treated with the Enterprise stent have been reported. It was hypothesized that endovascular intervention with the Enterprise stent for patients with posterior circulation atherosclerotic stenosis might be safe and efficacious, and this study was thus performed to investigate the safety and efficacy of the Enterprise stent in endovascular intervention of posterior circulation atherosclerotic stenosis.

## Materials and methods

### Subjects

**Fig.1:**
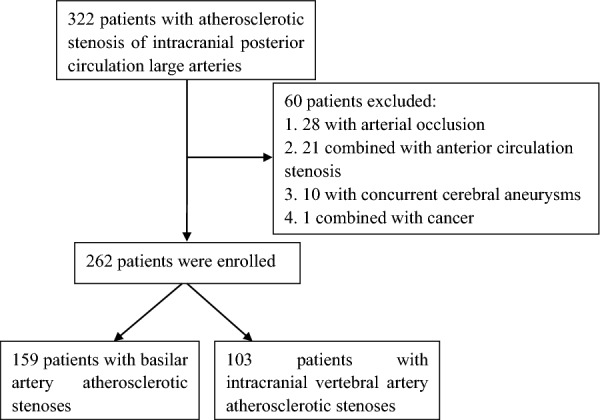
Flowchart of patient enrollment

This retrospective one-center study which was approved by the ethics committee of our hospital (with the ID number of 2020108) abided by the STROBE guideline, and no signed informed consent was needed from the patients because of the retrospective study design. Consecutive patients with ≥ 70% atherosclerotic stenosis of posterior circulation intracranial large arteries which was treated endovascularly with the Enterprise stent angioplasty between January 2019 and October 2021 were enrolled. The inclusion criteria were patients with ≥ 70% atherosclerotic stenosis of the posterior circulation intracranial large arteries confirmed by computed tomography angiography (CTA) or digital subtraction angiography (DSA), non-disabling stroke that left no or did not leave any significant disabilities after onset (including mild ischemic stroke and transient ischemic attack), ischemic cerebrovascular events which were caused by the relevant intracranial atherosclerotic stenosis and were refractory to optimal medications (post-medication symptoms remained including recurrent positional dizziness, blurred vision, dysarthria, ataxia, limb paralysis, impaired consciousness, or posterior circulation infarction confirmed by imaging), and an mRS score < 3 before enrollment for endovascular revascularization (Fig. 1). The exclusion criteria were patients with non-atherosclerotic occlusion of large posterior circulation arteries, severe stenosis of anterior circulation, allergies to heparin, aspirin, clopidogrel, metal implants or anesthetics, intolerance to general anesthesia, comorbidity of malignant tumors, severe liver and renal failure with the glomerular filtration rate reduced to below 25% of the normal values, and an expected life span of less than 1 year.

### Endovascular procedure

Perioperative dual antiplatelet therapy was conducted with clopidogrel (75 mg, once a day) and aspirin (100 mg, once a day) taken orally for ≥ 3 days before endovascular treatment. After successful sheath insertion into the right femoral artery, 50 IU/kg unfractionated heparin was injected by intravenous bolus shot for systemic heparinization, and 1000 IU unfractionated heparin was added every hour during the procedure.

The procedure was performed with the patient in the supine position under general anesthesia. After successful puncture of the right femoral artery using the improved Seldinger technique, a 6F 90 cm-long sheath was inserted before a 5F 115 cm-long intermediate catheter or a 6F guide catheter was navigated to the vertebral artery V3 segment. After angiography, the arterial diameter proximal and distal to the lesion was measured. Under road mapping, an SL-10 microcatheter (Stryker, USA) was sent to a location distal to the stenosis under the guidance of a 200 cm-long 0.014 inch (0.356 mm) Synchro micro-guide wire (Stryker, USA). After angiographic confirmation of the microcatheter within the arterial true lumen, an ASAHI 300 cm-long micro-guide wire whose distal end was shapped like a “pigtail” was exchanged into the artery. Then, a Gateway (Boston Scientific, Boston, MA, USA) balloon with a diameter of less than 80% of the normal vessel diameter was navigated along the micro-guide wire to the stenosis for pre-expansion at 6 Atm (609.275 kPa) for 10–15 s. After expansion, an Enterprise (Codman, USA) stent with a length of 3–5 mm exceeding both ends of the stenosis was introduced along a Prowler-21 stent catheter to the stenosis and released to fully cover the lesion. If the residual stenosis was greater than 40%, a balloon was used for expansion until the residual stenosis was ≤ 30%.

### Post-embolization management

Cranial CT examination was performed immediately after the procedure, blood pressure was strictly controlled below 140/90 mmHg, and patients were instructed to stay in bed with monitoring of the neurological and hemodynamic functions for at least 3 days. After the procedure, stable intravenous infusion of antihypertensive agents was performed to prevent and treat hyperperfusion. Clopidogrel (75 mg, once a day) combined with aspirin (100 mg, once a day) was used for standardized antiplatelet aggregation therapy for ≥ 3 days. Usually, the patient was discharged 5 days later, and clopidogrel and aspirin in the same doses were administered for 6 months. According to the recheck results of angiography, aspirin alone with the dose of 100 mg/daily was administered for a long time or lifelong use.

### Follow-up and parameters for evaluation

**Fig. 2:**
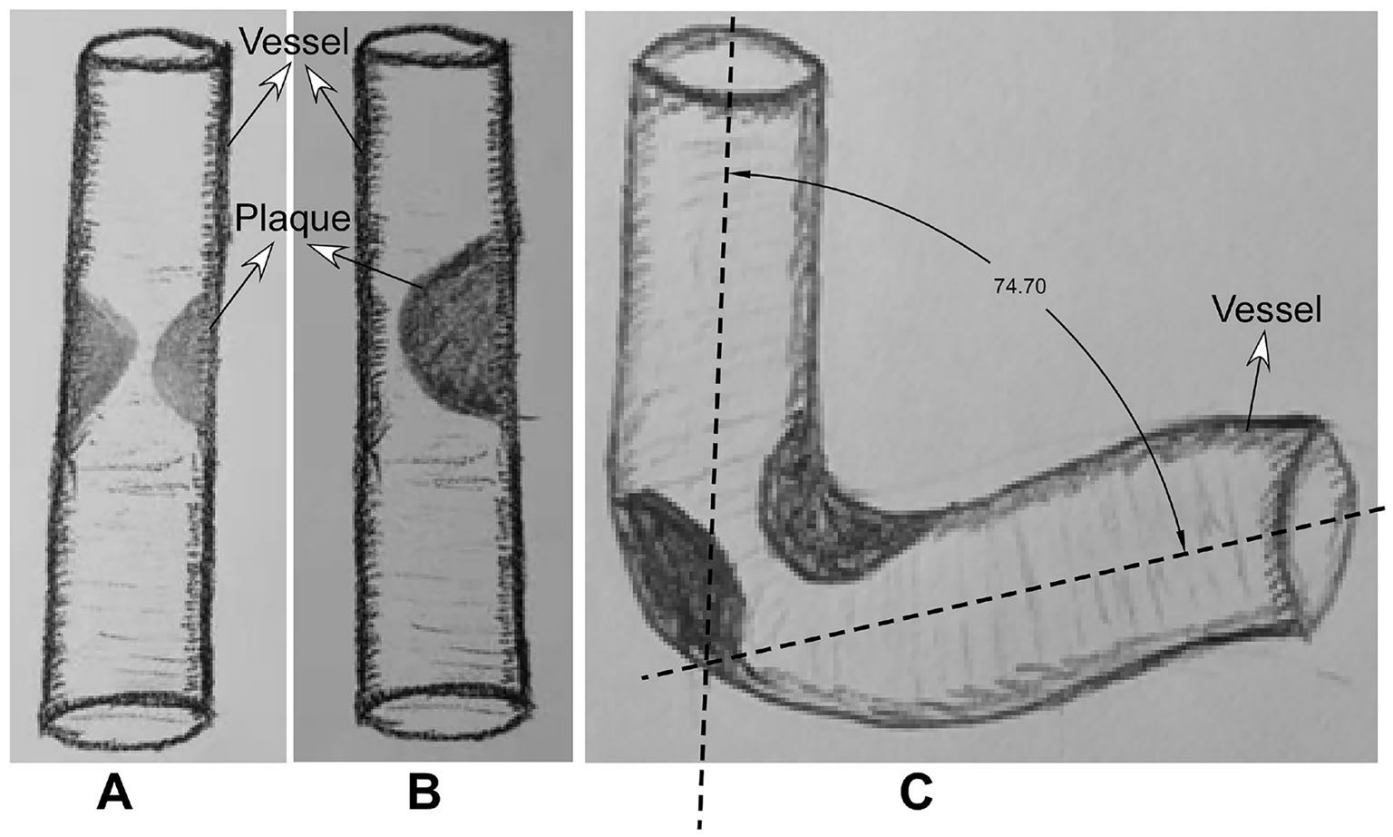
Atherosclerotic plaque morphology and measurement of the stenotic angle. **A**, **B**. The stenotic plaque morphology was centripetal (**A**) and eccentric (**B**). **C**. Measurement of the stenotic angle formed between the proximal and distal segments of the artery at the stenotic lesion. In this case, the stenotic angle was 74.70 degree

Follow-up was conducted by telephone, outpatient or inpatient reexamination. The clinical follow-up time was 30 days, 6 months, 1 year and 2 years after the embolization operation, and DSA or CTA was performed in 6–12 months. All neurological and non-neurological complications were recorded. In patients with no symptoms, the follow-up was performed once every 6 months. The patients were scored with the modified Rankin scale (mRS) before and after stent angioplasty and during follow-up. For efficacy evaluation, good outcomes were defined by the mRS score ≤ 2 at follow-up [22, 23]. Arterial or in-stent restenosis was defined as the stenosis ≥ 50% or the stenosis was increased by 30%. The following parameters were analyzed: age, sex, past history, hypertension, diabetes mellitus, coronary heart disease, hyperlipidemia, Hyperhomocysteinemia (HCY), smoking, alcohol abuse, symptoms, stenosis location, degree and length, atherosclerotic plaque morphology (Fig. 2), angles formed between the proximal and distal segments of the artery (Fig. 2), presenting and poststenting mRS, residual stenosis, complications within 30 days, 1 year stroke or death, and in-stent restenosis.

### Endpoint evaluation

The primary endpoint was defined as any new stroke (including hemorrhagic and ischemic) or death within 30 days after surgery and at 1 year, including stroke or TIA in the responsible area of the surgical vessel. The secondary endpoint was in-stent restenosis or occlusion. If the neurological deficit caused by hemorrhage or ischemia was more than 24 h, it was considered as a stroke. A new stroke needed to be confirmed by head CT or MRI. If the imaging manifestation was subarachnoid hemorrhage, cerebral parenchyma hemorrhage or intraventricular hemorrhage, it was considered as a hemorrhagic stroke regardless of symptoms. The in-stent restenosis was defined as ≥ 50% confirmed by DSA or CTA.

### Statistical analysis

The SPSS 22.0 software (IBM, Chicago, IL, USA) was used for data processing. Measurement data of non-normal distribution were represented by median and quartile [M (P25, P75)] and tested with the Chi square test. Measurement data of the normal distribution were presented by mean ± standard deviation and tested with the paired t-test. Categorical data were expressed as frequency and percentages. Univariat and mutivariat analyses were performed to investigate risk factors for periprocedural complications, 1 year stroke or death events, and in-stent stenosis with the odds ratio being calculated. Parameters with the P value ≤ 0.1 in the univariat analysis was entered in the mutivariat analysis to investigate the significant independent risk factors. Receiver operating characteristic (ROC) curve analysis was performed for continuous measurement data in the multivariate analysis. P < 0.05 meant significant differences.

## Results

### Subjects

Among 322 patients with symptomatic intracranial atherosclerotic stenosis in the posterior circulation who underwent Enterprise stenting from September 2019 to February 2022, 262 patients met the inclusion criteria and were enrolled (Fig. 1 and Table 1). These patients had a mean age of 60.9 ± 9.6 years old, including 202 male patients (77.1%). Hypertension was present in 199 cases (63.4%), diabetes mellitus in 97 cases (37.0%), coronary heart disease in 38 cases (14.5%), hyperlipidemia in 58 cases (22.1%), and hyperhomocysteinemia in 35 cases (13.4%). TIA was present in 133 cases (50.8%), cerebral infarction in 129 cases (49.2%), basilar artery stenosis in 159 cases (60.7%), and intracranial vertebral artery stenosis in 103 cases (39.3%). A total of 275 stenotic lesions were present, including a tandem stenosis in 13 (4.96%) patients. The mRS score was 0–4 (mean 1.32 ± 0.68 or median 1 and upper and lower quartiles (1, 1)), with 0 in 5 (1.9%) patients, 1 in 193 (73.7%), 2 in 43 (16.4%), 3 in 18 (6.9%), and 4 in 3 (1.1%). The length of stenosis ranged 2.3–23 mm (mean 6.3 ± 2.9), and the arterial angle formed at the stenosis was 81–180º (mean 156.4º ± 18.1º).

**Table 1:** Data and stratification analysis of the patients

	Total (n = 262)	BA (n = 159)	VA (n = 103)	P
Age				
	33–86 (60.6 ± 9.7)	33–86 (60.8 ± 9.7)	36–85 (60.2 ± 9.8)	0.586
Sex				
M	202 (77.1%)	118 (74.2%)	84 (81.6%)	0.167
F	60 (22.9%)	41 (25.8%)	19 (18.4%)	0.167
Past history				
Hypertension	199 (63.4%)	118 (74.2%)	81 (78.6%)	0.413
Diabetes	97 (37.0%)	58 (36.5%)	39 (37. %9)	0.820
Coronary heart disease	38 (14.5%)	23 (14.5%)	15 (14.6%)	0.982
Hyperlipidemia	58 (22.1%)	32 (20.1%)	26 (25.2%)	0.330
HHCY	35 (13.4%)	22 (13.8%)	13 (12.6%)	0.778
Smoking				
Total	127 (48.5%)	75 (47.2%)	52 (50.5%)	0.600
Past	65 (24.8%)	40 (25.2%)	25 (24.3%)	0.871
Current	62 (23.7%)	35 (22.0%)	27 (26.2%)	0.435
Drinking				
Total	100 (38.2%)	61 (38.4%)	39 (37.9%)	0.935
Past	45 (17.2%)	29 (18.2%)	16 (15.5%)	0.571
Current	55 (21.0%)	32 (20.1%)	23 (22.3%)	0.669
Symptoms				
TIA	133 (50.8%)	75 (47.2%)	58 (56.3%)	0.135
Infarction	129 (49.2%)	84 (52.8%)	45 (43.7%)	0.135
Stenotic degree				
Total	70–99% (87.2 ± 5.9%)	70–99% (87.9 ± 6.4%)	70–99% (85.8 ± 10.1%)	0.350
70–80%	72 (27.5%)	41 (25.8%)	31 (30.1%)	0.445
80–90%	102 (38.9%)	68 (42.8%)	34 (33.0%)	0.114
90–99%	88 (33.6%)	50 (31.4%)	38 (36.9%)	0.362
Prestenting				
mRS	0–4 (1.32 ± 0.68)	1–4 (1.3 ± 0.63)	0–4 (1.3 ± 0.75)	0.618
Stenotic length (mm)	2.3–23 (6.3 ± 2.9)	2.5–18 (6.1 ± 2.6)	2.3–23 (6.7 ± 3.4)	0.106
Stenotic angle(º)	81–180 (156.4 ± 18.1)	81–180 (158.0 ± 18.2)	85–180 (153.9 ± 17.9)	0.074
Poststenting				
Residual stenosis	5–40% (19.2 ± 6.2%)	5–40% (19.2 ± 6.2%)	5–40% (19.2 ± 6.3%)	0.919
30 days–complications	14 (5.3%)	12(7.5%)	2 (1.9%)	0.049
Follow-up				
30 days–1 year stroke or death	7 (2.9%)	3 (2.0%)	4 (4.1%)	0.348
In-stent restenosis	35 (15.7%)	12 (8.8%)	23 (26.4%)	0.000

Data were presented as mean ± standard deviation or frequency and percentages

*HHCY* Hyperhomocysteinemia, *TIA* transient ischemic attack, *BA* basilar artery, *VA* intracranial vertebral artery, *mRS* modified Rankin scale score

### Endovascular stent angioplasty

Stent angioplasty was successful in all patients, with 267 Enterprise stents being implanted and two stents deployed in each of five patients, including 104 (39.0%) 4 mm-diameter stents and 163 (61.0%) 5 mm-diameter stents with a stent length ranging from 14 to 30 mm. A balloon-expanding stent was also deployed in eight patients. The stenosis degree was reduced from 86.3 ± 6.2% before stenting to 19.3 ± 5.4% after stenting. Complications occurred in 14 (5.3%) patients, including perforator infarction in 7 patients who were improved after treatment, in-stent thrombosis in two patients (one case was treated with successful emergency recanalization and had 2 mRS points half a year later, and the other case had 5 mRS points half a year later after failed emergency recanalization), and dissection in two patients (one had no symptoms and the other experienced serious infarction and was bedridden with an mRS of 5 half a year later). One patient experienced thalamic ventricular hemorrhage and died 3 months later after conservative treatment, one had subarachnoid hemorrhage which was improved after conservative treatment (2 mRS points half a year), and one patient experienced postoperative pulmonary infection which was improved by symptomatic support treatment (mRS 1 half a year later). The complication rate was 1.9% (2/103) in the intracranial vertebral artery, which was significantly (Z = 3.883, P = 0.049) greater than that in the basilar artery (7.5% or 12/159).

### Follow-up

Clinical follow-up was performed in 245 (93.51%) patients for a mean duration of 16.5 ± 7.3 months, with more than 24 months in 31 (11.83%) patients, 18–24 months in 72 (27.48%), and 12–18 months in 142 (54.20%). At half a year, the mRS score was 0–6 [mean 0.44 ± 0.96 or median 0 and upper and lower quartiles (1, 0)] which was significantly (P < 0.0001) better than that before stenting (mean 1.32 or median 1), with 0 in 175 (71.4%) patients, 1 in 53 (21.6%), 2 in 8 (3.3%), 3 in 2 (0.8%), 4 in 3 (1.2%), 5 in 2 (0.8%), and 6 in 2 (0.8%), with good outcomes of mRS ≤ 2 in 236 (96.3%) and poor outcomes (mRS score > 2) in 9 (3.7%). At 1 year follow-up, 7 patients (2.9%, 7/245) had recurrent symptoms, including 4 patients with stenting for the vertebral artery stenosis and 3 with stenting for the basilar artery stenosis. Three patients experienced dizziness and unclear vision, 3 patients exhibited cerebral infarction, and 1 patient experienced cerebral hemorrhage (Table 1).

Imaging follow-up was conducted in 223 (85.11%) patients 6–33 months (mean 15.3 ± 5.2) after embolization, including DSA in 154 (58.78%) patients and CTA in 69 (26.34%). In-stent restenosis was present in 35 patients (15.7%, 35/223), with the restenosis rate of 26.4% (23/87 or 23 patients) in the stented intracranial vertebral artery, which was significantly (Z = 12.440, P < 0.001) greater than that in the stented basilar artery (8.8%, 12/136, or in 12 patients). Six patients (2.7%) with in-stent restenosis were symptomatic.

Risk factors for periprocedural complications, residual stenosis after stenting, 1 year stroke or TIA, and in-stent restenosis were analyzed with the univariate logistic regression analysis (Table 2). While no significant (P > 0.05) risk factors were found for residual stenosis, diabetes mellitus (χ^2^ = 7.06, P = 0.008), coronary heart disease (χ^2^ = 4.17, P = 0.041), and stenotic location (basilar or vertebral artery, χ^2^ = 4.33, P = 0.038) were significant (P < 0.05) risk factors for periprocedural complications. Stenotic length (χ^2^ = 7.98, P = 0.004), plaque morphology (χ^2^ = 5.98, P = 0.015), and use of tirofiban (χ^2^ = 9.19, P = 0.0024) were significant (P < 0.05) risk factors for the 1 year stroke or death rate, whereas high HCY (χ^2^ = 9.28, P = 0.002) and stenotic length (χ^2^ = 4.78, P = 0.029) were significant risk factors for the in-stent stenosis rate. Mutivariate analysis revealed that stenotic length was the only significant (P = 0.026 and 0.024, respectively) independent risk factor for 1 year stroke or death events and in-stent restenosis (Table 2).

**Table 2:** Univariat and multivariate analyses of risk factors

Dependent factors	Independent factors	Univariate analysis			Multivariate analysis	
		OR 95% CI	Statistics	P	Statistics	P
Periprocedural complications	Diabetes	4.55 (1.39–14.92)	7.06	0.008		
	Coronary heart disease	3.62 (1.14–11.47)	4.17	0.038		
	Stenosis location at BA	4.05 (1.21–18.50)	4.33	0.38	4.88	0.027
1 year stroke or death rate	Stenotic length > 8 mm	9.45 (1.79–50.16)	7.91	0.005	4.93	0.026
	Plaque morphology	6.98 (1.32–36.91)	5.98	0.015		
	Use of tirofiban	11.40 (2.14–60.70)	9.19	0.002	7.06	0.008
In-stent restenosis	High HCY	3.66 (1.60–8.35)	9.28	0.002	7.03	0.008
	Stenotic length > 8 mm	2.46 (1.03–5.83)	4.78	0.029	5.12	0.024

*OR* odds ratio, *CI* confidence interval, *BA* basilar artery, *HCY* Hyperhomocysteinemia

**Fig. 3:**
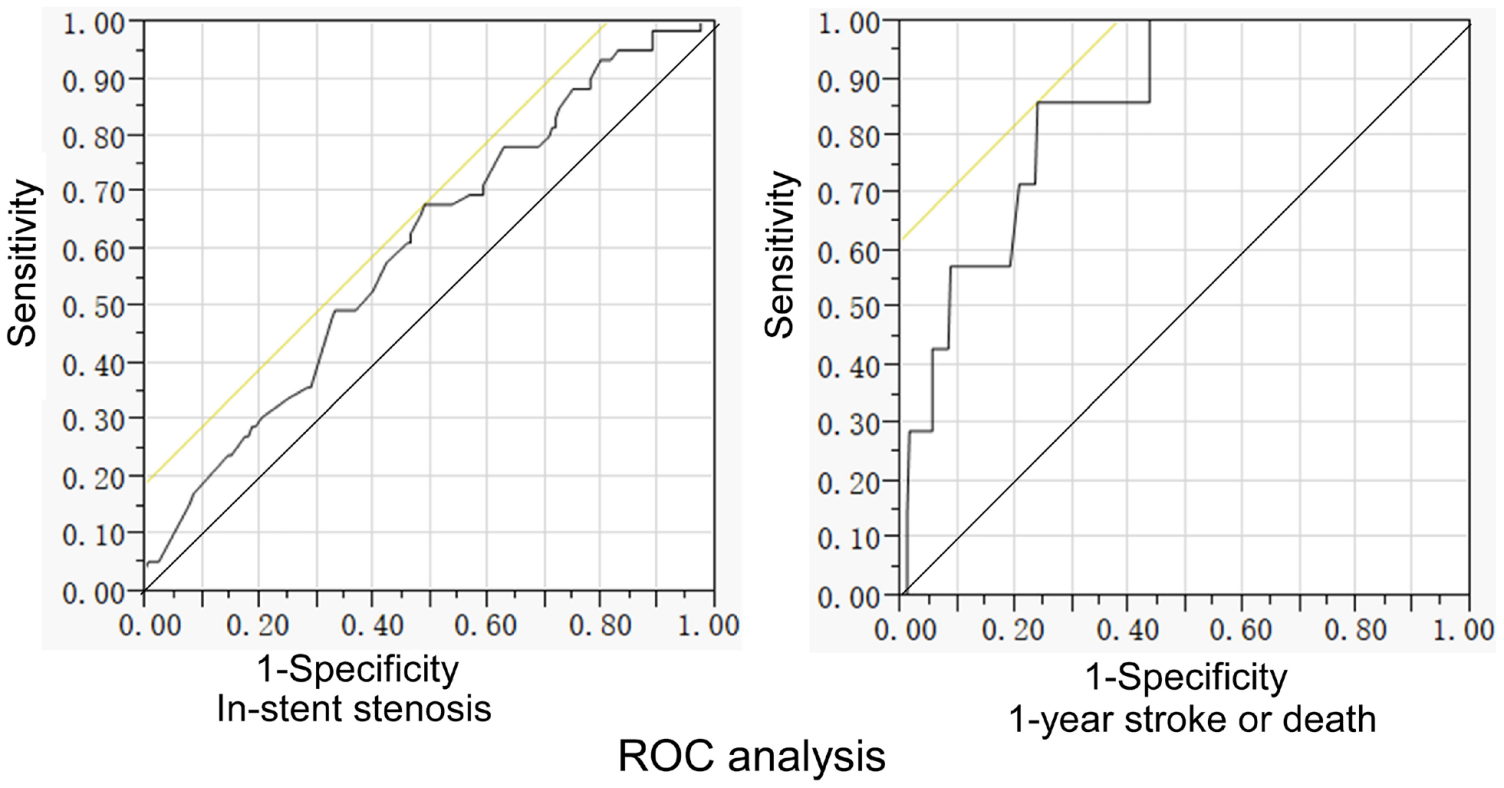
Receiver operating characteristics (ROC) curve analysis for in-stent stenosis and 1-year stroke or death

ROC curve analysis revealed that the stenotic length had a cutoff value of 5.7 mm with an area under the curve (AUC) of 0.61, a sensitivity 0.68, a specificity 0.51, positive predictive value (PPV) 33.1%, and negative predictive value (NPV) 81.6% for the in-stent restenosis. For 1 year stroke or death events, the stenotic length had a cutoff value of 7.7 mm, with an AUC of 0.85, a sensitivity 0.86, a specificity 0.76, a PPV 9.1%, and a NPV 99.5% (Table 3 and Fig. 3).

**Table 3:** ROC analysis of stenotic length for in-stent restenosis and 1 year stroke or death

Independent variable	Dependent variable	Cutoff (mm)	AUC	Sensitivity	Specificity	PPV (%)	NPV (%)
Stenotic length	In-stent restenosis	5.7	0.61	0.68	0.51	33.1	81.6
	1 year stroke or death	7.7	0.85	0.86	0.76	9.1	99.5

*ROC* receiver operating characteristics, *AUC* area under the curve, *PPV* positive predictive value, *NPV* negative predictive value

## Discussion

In this study, it was found that the Enterprise stent could be safely and efficaciously applied in the treatment of symptomatic severe posterior circulation atherosclerotic stenosis, with a high success rate of stenting, a relatively low rate of in-stent restenosis at follow-up, and a low rate of recurrent stroke within 1 year.

The prevalence of intracranial atherosclerotic stenosis in China is as high as 46.6%, and patients who are refractory to conservative treatment often need further endovascular intervention [24]. Endovascular intervention can achieve good outcomes with decreased peri-procedural complication rates and increased treatment effects if appropriate patients have been selected and endovascular interventional skills have been improved [11–13].

The perforators of posterior circulation vertebrobasilar arteries are abundant, and the risk of postoperative complications is relatively high. The Wingspan stent has a hard tip and can easily cause vascular injury and postoperative restenosis [25]. This requires not only strict adherence to relevant indications and selection of experienced operators, but also use of good flexible stents to reduce complications. Compared with the Wingspan stent, the Enterprise stent has better flexibility and smaller radial force, which is beneficial to reducing perioperative complications [26]. The Enterprise stent is of closed-cell design, easier to operate, and easily delivered through microcatheters. They can be retrieved when the release rate is less than 70%, which makes the release more accurate. In addition, a softer flexible delivery microcatheter can be used to deliver the Enterprise stent through tortuous vessels to smaller terminal vessels [26].

The peri-procedural complication rate of endovascular therapy for posterior circulation atherosclerotic stenosis is higher than that of the anterior circulation. In a Meta-analysis of 1177 cases of intracranial atherosclerotic stenoses treated with stents, the complication rates of stenting in the anterior and posterior circulation were 6.6% and 12.1%, respectively [27]. The rate of perioperative ischemic events in the distribution area of perforating branches in the anterior and posterior circulation has been reported to be 14.5% in the posterior circulation versus 5.1% in the anterior circulation, and the rate of recurrent stroke in the posterior circulation was 10.4% vs 3.4% in anterior circulation [18]. The higher complication rate after posterior circulation stent implantation may be related to the rich perforator vessels of the basilar artery, and the “snowplow effect” caused by balloon dilation and stent implantation is prone to perforator cerebral infarction [28]. In our study, the success rate of stent angioplasty was 100%, the incidence of perioperative complications within 30 days was 5.3%, and the 1 year recurrent stroke rate was 2.9%, which was significantly lower than those of the SAMMPRISE [9] and the VISSIT study [10]. In the SAMMPRISE study [9], the 30 day rate of stroke or death was 14.7% in the stenting group and 5.8% in the medication group, and the 1 year rate of primary end point was 20.0% in the stenting group and 12.2% in the medication group. In the VISSIT trial [10], the 30 day primary safety end point was 24.1% in the stent group and 9.4% in the medical group, and the 1 year primary outcome of stroke or hard TIA was 36.3% in the stent group and 15.1% in the medical group. The better outcomes achieved in our study are probably attributed to the use of balloons with a diameter < 80% of the normal arterial diameter in expanding the stenoses and the Enterprise stent with relatively weak radial force for stent angioplasty. Our outcomes are similar to those of the CASSISS study with a complication rate of 5.1%, and a 1 year recurrent stroke rate of 2.8% [13]. Another study also reported a low incidence of recurrent ischemia related to the stented artery of 2.2% during 10.2 months of mean follow-up after angioplasty treatment with the Enterprise stent [20].

The postoperative restenosis rate was 17.6–26.5% after the Wingspan stent angioplasty for intracranial stenosis [10–12] because the high radial force of the Wingspan stents may contribute to significant intimal hyperplasia [19]. The restenosis rate of the Enterprise stent had been reported from 3.3 to 24.7% [29], and the incidence of symptomatic restenosis was 9.3% [20]. In a study including 35 patients with symptomatic severe basilar atherosclerosis stenosis treated with the Enterprise stent, the incidence of restenosis was reported to be 17.6% at 6 month follow-up [21]. In addition, in a study enrolling 44 patients with symptomatic severe intracranial atherosclerotic stenosis treated with the Enterprise stent, the average incidence of restenosis at follow-up of 25.6 months was 6.81% [30]. In our study, 35 patients (15.7%, 35/223) experienced in-stent restenosis, and 6 patients (2.7%) had symptomatic in-stent restenosis at follow-up, which are better than those with the Wingspan stent. The closed-cell design of the Enterprise stent may contribute to hemodynamic changes and subsequent intimal reaction by altering the shape of the artery more than the Wingspan stent. Moreover, better flexibility and small radial force of the Enterprise stent may also be able to reduce the intimal hyperplasia and in-stent restenosis.

Subgroup analysis of our study found that the perioperative complication rate in stenting the intracranial vertebral artery was 1.9%, which was significantly lower than that of stenting the basilar artery (7.5%). However, the in-stent restenosis rate in the intracranial vertebral artery stenting was 26.4%, which was significantly higher than that in the basilar artery stenting (8.8%). Compared with the basilar artery which harbors rich perforating vessels, the vertebral artery does not have so many perforators, and the probability of procedural perforating complications in the vertebral artery is low [7]. This is related to the anatomical characteristics and hemodynamic instability of the vertebral artery V4 segment. The structure of intracranial artery wall is different from that of other parts of the human body. The intracranial arterial wall has a thick intima and a well-developed elastic membrane, whereas the middle and outer walls are thin without an elastic membrane [31]. The intracranial vertebral artery is a transitional part from the extracranial to the intracranial artery, and has the characteristics of both the extracranial and intracranial arteries [31]. The main pathological mechanism of in-stent restenosis is intimal hyperplasia [32]. Endometrial reaction may be different between the extracranial and intracranial arteries, which is worthy of further study. In our study, the restenosis of the Enterprise stent is relatively high, especially in stenting the intracranial vertebral artery, which may be related to the specific arterial structure as stated above even though detailed investigation is necessary to reveal the true reason for this.

The Winspan stent is very common [9, 10, 28], but the release process of this stent is complex. For complex lesions such as angulated and tortuous stenoses, it is very difficult for the Winspan stent to pass through. The distal olive end of the stent is hard and can easily cause vascular damage, especially the tip of the basilar artery [25]. For stenoses in the middle and upper segments of the basilar artery, the complication rate may be high if the Wingspan stent is deployed [10, 16–18]. In order to decrease the complications of angioplasty for arterial stenoses, the Enterprise stent is used to treat the arterial stenosis in our study because of the higher safety of the Enterprise stent.

In logistic regression analysis, our study found that diabetes mellitus, coronary heart disease, and stenotic location at the basilar artery were significant (P < 0.05) risk factors for periprocedural complications. Stenotic length, plaque morphology, and use of tirofiban were significant risk factors for the 1 year stroke or dearth rate, whereas high HCY and stenotic length were significant risk factors for the in-stent stenosis rate. Nonetheless, the stenotic length was the only significant (P < 0.05) independent risk factor for 1 year stroke or death events and in-stent restenosis. With increase of the stenotic length, a longer stent is needed and may lead to increased risk of in-stent thrombosis, restenosis and subsequent stroke. After studying the long-term recurrence rate of stroke in a cohort of symptomatic patients of intracranial atherosclerotic stenosis treated endovascularly, Zhang et al. found that the posterior circulation stenosis was an independent risk factor for recurrence of stroke while good antegrade flow (TICI 3/4) could lower the recurrence risk in the posterior circulation stenosis after endovascular treatment [2].

Some limitations existed in our study, including the retrospective one-center study design, Chinese patients enrolled only, no randomization or control, which may all affect the outcomes of the study. Future prospective, multi-center, randomized, controlled clinical trials involving multiple races and ethnicities may be able to resolve all these issues for better outcomes.

In conclusion, the Enterprise stent can be safely and efficaciously applied in the treatment of symptomatic severe posterior circulation atherosclerotic stenosis, with a high success rate of stenting, a relatively low rate of in-stent restenosis at follow-up, and a low rate of recurrent stroke within 1 year.

## Data Availability

The data and materials are available from the corresponding author on reasonable request.
